# 178. Impact of a microbiology comment nudge on the diagnostic evaluation of *Staphylococcus aureus* bacteriuria

**DOI:** 10.1093/ofid/ofac492.256

**Published:** 2022-12-15

**Authors:** Dimple Patel, Aiman Bandali, Pamela Giordano, Jason Kessler, Robert Roland

**Affiliations:** Atlantic Health System, Morristown, New Jersey; Atlantic Health System, Morristown, New Jersey; Atlantic Health System, Morristown, New Jersey; Morristown Medical Center, Morristown, New Jersey; Overlook Medical Center, Summit, New Jersey

## Abstract

**Background:**

*Staphylococcus aureus* is a relatively uncommon cause of urinary tract infections. *S. aureus* bacteriuria is thought to result most commonly from urinary tract instrumentation (ascending infection) or via hematogenous seeding of the genitourinary tract (descending infection). Given the devastating impact of invasive *S. aureus* infection and awareness that *S. aureus* bacteriuria is often a marker for *S. aureus* bacteremia, growth of *S. aureus* from urine cultures should prompt urgent clinical assessment, including blood culture collection. In April 2021, a comment nudge was added to the microbiology culture and susceptibility report for all *S. aureus* isolated from urine cultures performed at the Atlantic Consolidated Laboratory (ACL) at Atlantic Health System. The purpose of this study is to assess the impact of a comment nudge on the diagnostic evaluation of *S. aureus* bacteriuria.

S. aureus bacteriuria comment nudge

**Figure:**
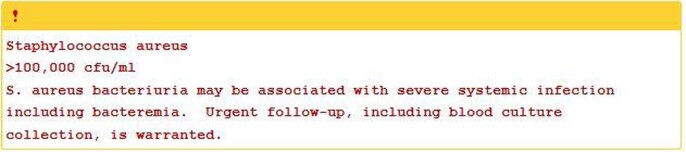

**Methods:**

All patients with *S. aureus* bacteriuria were retrospectively identified from the ACL microbiology database. The primary endpoint was collection of blood cultures within 96 hours of positive urine culture during the 6-month period pre-comment (October 2020 – April 2021) compared to 6-month period post comment (May 2021 – November 2021). In addition, incidence of *S. aureus* bacteremia among the patients with collected blood cultures was also evaluated.

**Results:**

*S. aureus* bacteriuria was identified in 122 patients during the Pre-Comment period and 188 patients in the Post-Comment period. Blood cultures were collected more frequently in the Post-Comment period compared to the Pre-Comment period (64% vs. 51%, p=0.018). Among patients for whom blood cultures were collected, 29% had concomitant *S. aureus* bacteremia in the Pre-Comment period compared to 16% in the Post-Comment period (p=0.03).

**Conclusion:**

The addition of a comment nudge to the microbiology report of urine cultures growing *S. aureus* significantly improved follow-up blood culture collection. Health systems may consider implementation of similar interventions, particularly in resource-limited settings, to avoid delayed recognition of *S. aureus* bacteremia.

**Disclosures:**

**All Authors**: No reported disclosures.

